# Broadening the reach of *Health Expectations*

**DOI:** 10.1111/hex.12533

**Published:** 2017-01-04

**Authors:** Carolyn A. Chew‐Graham

**Affiliations:** ^1^ Research Institute, Primary Care and Health Sciences, Keele UniversityStaffordshireUK; ^2^ South Staffordshire and Shropshire Healthcare NHS Foundation TrustStaffordshireUK

The Editorial team at *Health Expectations* view our journal as one with international appeal and reach. We pride ourselves on being receptive to manuscripts from around the world and value the work of our international colleagues. Reflecting on the submissions received in 2016, we note that whilst most manuscripts came from the UK, the USA, the Netherlands and Australia, the breadth of countries from which manuscripts reporting primary research was huge, including China, Peru, Nigeria, Romania, Taiwan and Thailand. The Associate Editors (themselves from the UK, Canada and Greece) note that the level of patient and public engagement (PPIE) in research varies in different parts of the world, and it is often because of the lack of PPIE in studies that manuscripts are not deemed suitable for HEX.

Introducing this issue of HEX, I note with some dismay that none of the manuscripts originate from developing countries, and this may limit the appeal of HEX in these countries.

So, if you are a researcher reading this Editorial briefing, conducting research, in developing countries, in which public participation in health care and health policy is the focus, we do want to encourage you to submit your manuscript to us.

On average, HEX receives 300 submissions per year which are processed by the Editorial team, and we accept about 30% of manuscripts. This is quite a high workload for our team of four, and we are seeking applications to fill an additional Associate Editor (AE) post. The AE role is vital in ensuring that we can maintain our excellent average turnaround time from submission to first decision of 32 days. So, if you already have experience reviewing manuscripts for journals (particularly HEX) and would like to join the team of Associate Editors, please contact us HEXedoffice@wiley.com.

Along with the Associate Editors, we have a strong Editorial Board, but again, reflecting on the membership, the Board would be strengthened with Board members from developing countries. Editorial Board members are particularly important in making editorial decisions when we have conflicting reviews, and the Associate Editor looks to a Board member for advice. If you are reading this and interested in becoming a member of the Board, please contact us.

Returning to this edition of HEX, I have decided to highlight manuscripts that I think will have an appeal internationally. Protheroe et al report a face‐to‐face survey conducted in Stoke‐on‐Trent, a deprived area of England. The survey used a measure of functional health literacy, the “Newest Vital Sign” (NVS), validated for use in a UK population. The study team, a partnership between academics and the City Council, demonstrated associations between higher rates of limited health literacy and older age, lower educational level, lower income, perceived poor health and lack of access to the Internet. We are pleased that the authors chose to publish this work in HEX— it is the first citywide survey of health literacy levels conducted in the UK. The authors emphasise that the evidence generated from their study, which is locally extremely relevant, will provide a key impetus to multidisciplinary, multisector collaborations and result in directly relevant important interventions to improve the public health of the city. The relevance of this study to international researchers and public health physicians is obvious, and we expect that this paper will be highly cited in academic journals and used to inform future public healthdevelopments.

The viewpoint article by Armstrong and colleagues proposes a ten‐step framework outlining steps and options for patient engagement in guideline development and highlights methods by which this can be achieved. The proposed framework could serve as a resource for guideline developers wishing to increase patient engagement at different steps of the practice development life cycle. Again, it is a key message not just for readers in the USA, Armstrong's home country, but around the world.

Carter‐Harris et al report a qualitative study exploring the views of people who smoke about lung cancer screening. They highlight factors such as stigma associated with smoking and distrust of medics which impact on uptake of screening procedures and engagement with medical care. The authors stress the need for patient‐centred information about lung cancer screening and for health‐care providers to recognize an individual's circumstances and preferences. The relevance of this work for other conditions where stigma might be a factor is not difficult to see. Robotin and colleagues report the development of “user‐friendly patient resources” for people affected with liver cancer, which complement health‐care provider information and support informed patient decision making. In their review of existing information and resources, they noted the lack of accessible information for people of low literacy. Using qualitative methods to explore perspectives of patients from different cultural backgrounds, they then developed resources that are available in four languages, as separate modules, and accessible online and in DVD format. The study team's recognition of the importance of patients' levels of health literacy in the provision of information resonates with Protheroe's paper. From Stoke‐on‐Trent to New South Wales, the issues are the same.

This edition also emphasises the growing role of technology in the provision of health care and health care interventions: Kendall et al report how young people access help and support using an on‐line forum, whilst Shulver and colleagues used qualitative methods to explore acceptability of a home tele‐rehabilitation programme to older adults. These studies reflect the growing trend to the development of interventions delivered using technology. What must not happen is that for people who do not have internet access (and may have poor health literacy) to be excluded from such interventions. Thus, it is vital that there is patient and public input into the development of such interventions, as demonstrated by Robotin and her team.

As usual, we present a variety of manuscripts in different clinical areas, utilising different research methods and with key messages that should appeal to our broad readership. If you would like to help shape the content of HEX by becoming an Associate Editor, then do please contact us. If you want to see your research published in HEX, our message is that we encourage submissions from around the world, so we look forward to receiving your submission.

